# Distribution of tick-borne diseases in China

**DOI:** 10.1186/1756-3305-6-119

**Published:** 2013-04-23

**Authors:** Xian-Bo Wu, Ren-Hua Na, Shan-Shan Wei, Jin-Song Zhu, Hong-Juan Peng

**Affiliations:** 1 Department of Epidemiology, School of Public Health and Tropical Medicine, Southern Medical University, Guangzhou, Guangdong, 510515, China; 2 Department of Pathogen Biology, School of Public Health and Tropical Medicine, Southern Medical University, Guangzhou, Guangdong, 510515, China; 3 Department of Biochemistry, Virginia Tech, 311 Engel Hall, Blacksburg, VA, 24061, USA

**Keywords:** Ticks, Tick-borne diseases, Epidemic, China

## Abstract

As an important contributor to vector-borne diseases in China, in recent years, tick-borne diseases have attracted much attention because of their increasing incidence and consequent significant harm to livestock and human health. The most commonly observed human tick-borne diseases in China include Lyme borreliosis (known as Lyme disease in China), tick-borne encephalitis (known as Forest encephalitis in China), Crimean-Congo hemorrhagic fever (known as Xinjiang hemorrhagic fever in China), Q-fever, tularemia and North-Asia tick-borne spotted fever. In recent years, some emerging tick-borne diseases, such as human monocytic ehrlichiosis, human granulocytic anaplasmosis, and a novel bunyavirus infection, have been reported frequently in China. Other tick-borne diseases that are not as frequently reported in China include Colorado fever, oriental spotted fever and piroplasmosis. Detailed information regarding the history, characteristics, and current epidemic status of these human tick-borne diseases in China will be reviewed in this paper. It is clear that greater efforts in government management and research are required for the prevention, control, diagnosis, and treatment of tick-borne diseases, as well as for the control of ticks, in order to decrease the tick-borne disease burden in China.

## Review

Ticks can carry and transmit viruses, bacteria, rickettsia, spirochetes, protozoans, *Chlamydia, Mycoplasma*, Bartonia bodies, and nematodes [1,2]. Approximately 10 genera of ticks, 119 species including 100 species of *Ixodidae* and 19 species of *Argasidae*, have been identified in China [1].

Tick-borne diseases are a major contributor to vector-borne diseases, and are distributed worldwide. They are mainly natural focal diseases, and most often occur in forests, bushes, and semi-desert grasslands. Globally, the number of distinct and epidemiologically important tick-borne diseases, including tick-borne encephalitis, Kyasanur forest disease, Crimean–Congo hemorrhagic fever, and Rocky Mountain spotted fever, has increased considerably over the last 30 years [3]. The vast territory, complex geography, and climate of China contribute to the abundance and diversity of ticks, thus tick-borne diseases are prevalent in most parts of China and seriously affect human health [1]. In recent years, tick-borne diseases have occurred in almost all Provinces/Autonomous Regions/Municipalities (P/A/M) in China and the infection rate continues to rise (Table 1) [2,4]. Continuous reports of emerging tick-borne disease cases in Shandong, Henan, Hebei, Anhui, and other provinces demonstrate the rise of these diseases throughout China (Table 1) [4]. Therefore, furthering knowledge of the epidemic status and the distribution of tick-borne diseases in China is extremely urgent for the prevention and control of these diseases, as well as for reducing the disease burden.

**Table 1:** The information of major tick-borne diseases reported in China

**Categories**	**Tick-borne diseases**	**Causative pathogens**	**Districts of endemic / case reported/population serological positive**	**Prevalence P/M/A number**	**Reference**
Zoonotic bacterial diseases	Lyme borreliosis	*Borrelia burgdorferi sensu lato*	Anhui, Beijing, Chongqing, Fujian, Gansu, Guangdong, Guangxi, Guizhou, Hebei, Heilongjiang, Henan, Hubei, Hunan, Inner Mongolia, Jiangsu, Jiangxi, Jilin, Liaoning, Ningxia, Shandong, Shaanxi, Shanxi, Sichuan, Tianjin, Tibet, Qinghai, Xinjiang, Yunnan, Zhejiang	29	[5-8]
	Q-fever	*Rickettsia burneti (Coxiella burnetii)*	Anhui, Beijing, Chongqing, Fujian, Gansu, Guangdong, Guangxi, Guizhou, Hainan, Hebei, Heilongjiang, Jiangsu, Jilin, Liaoning, Ningxia, Qinghai, Shandong, Shaanxi, Taiwan Outbreak in Inner Mongonlia, Sichuan, Xinjiang, Yunnan, Tibet	24	[7,9-11]
	tularemia	*Francisella tularensis*	Beijing, Heilongjiang, Inner Mongolia, Qinghai, Shandong, Tibet, Xinjiang,	7	[10,12,13]
	North-Asia tick-borne spotted fever	*Rickettsia sibirica, Rickettsia conorii, Rickettsia akari*	Beijing, Guangdong, Heilongjiang, Jilin, Liaoning Inner Mongolia, Xinjiang	7	[7,10]
	Oriental spotted fever	*Richettsia japonica*	Hainan	1	[14]
Zoonotic viral diseases	Tick-borne encephalitis (Forest Encephalitis)	tick-borne encephalitis virus	Liaoning, Jilin, Heilongjiang, Inner Mongolia, Xinjiang, Tibet, Yunnan, Sichuan, Hebei, Greater Khingan Range, Changbai Mountains, the Altai Mountains, Tianshan Mountain	9	[7,9,15-18]
	Crimean-Congo hemorrhagic fever (Xinjiang hemorrhagic fever)	Crimean-Congo hemorrhagic fever virus	Cases reported from Xinjiang and Junggar; serological evidence shown in Qinghai, Yunnan, Sichuan, Inner Mongolia, Anhui, Hainan and northeast Yili	7	[7,9,16]
	Colorado fever	Colorado virus	Cases reported in Beijing, Yunnan, Gansu, Hainan, Xinjiang	5	[7]
Emerging diseases	Novel Bunyavirus infection	Novel Bunyavirus	Jiangsu, Hubei, Henan, Shandong, Anhui, Liaoning, Zhejiang, Yunnan, Guangxi, Jiangxi , Shannxi	11	[19,20]
	Human monocytic ehrlichiosis	*Ehrlichia. canis Ehrlichia chaffeeusis*	Guangdong, Guangxi, Hunan, Liaoning, Jilin, Heilongjiang, Xinjiang	7	[21-23]
	Human granulocytic anaplasmosis	*Anaplasma phagocytophilum*	Anhui, Tianjin, Shandong, Heilongjiang, Inner Mongolia, Xinjiang,, Hainan	7	[21,23,24]
livestock parasitic diseases	Piroplasmosis	*Theileria luwenshuni Theileria uilenbergi Theileria sinense Babesia motasi*	Qinghai, Gansu, Ningxia, Sichuan, Yunnan	5	[25]
Spirochetosis	Tick-borne relapsing fever	*Borrelia persica*	Beijing, Guangdong, Heilongjiang, Jilin, Liaoning, Inner Mongolia, Xinjiang	7	[7,9]

### Common tick-borne zoonoses in China

#### Lyme borreliosis

Lyme borreliosis (LB), also called Lyme disease in China, is a natural focal disease caused by *Borrelia burgdorferi* sensu lato*.* LB usually manifests as an acute disease. It only becomes chronic in a small proportion of patients, if left untreated. It is named after Lyme, a town in Connecticut, US, where it was first discovered in 1975 [26]. Lyme borreliosis is widely distributed, and has been reported in more than 70 countries on five continents. Moreover, the affected area continues to expand and the incidence of this disease is on the rise [5]. It was first reported in China in 1985, in a forest region in Hailin County, Heilongjiang [27]. The peak of incidence of Lyme borreliosis appears to occur from June to August. Its main vectors are *Ixodes persulcatus* in Northern China,*Ixodes granulatus* and *Ixodes sinensis* in Southern China, and *Haemaphysalis bispinosa* ticks may act as the vector in Southern China [6,28]. Human cases of Lyme borreliosis have been confirmed in 29 provinces/municipalities. As demonstrated by its occurrence, its natural foci are present in at least 19 provinces/municipalities in China (Table 1). The major endemic areas in China are forests in the Northeast and Northwest and some areas in North China [29]. In Heilongjiang, Jilin, Liaoning, and Inner Mongolia, over 3 million people suffer tick bites annually, of those, approximately 30,000 people become infected with Lyme borreliosis; approximately 10% of the new cases may turn into chronic infections over 2 to 17 years without treatment [7]. It was reported that the serological positivity of LD was 1.06~12.8% in the 30,000 people randomly sampled (from approximately 20 P/A/M), with a mean positivity rate of 5.06% overall and 5.33% in the forests; the morbidity was 1.16~4.51% in the forests of Northeastern China, with a mean morbidity of 2.84% [29].

### Tick-borne Encephalitis

Tick-borne Encephalitis (TBE), also known as Forest Encephalitis in China, is an acute infectious disease of the nervous system caused by the TBE virus (TBEV). This virus was first isolated from patients using mouse inoculation by Tkachev in 1936 [30]. In China, TBE was first observed in 1942, and TBEV was first isolated from patients and ticks in 1952 [31]. Among the three subtypes of TBEV, the European, the Siberian, and the Far-Eastern subtype, the latter is endemic in North China and is also present in Western and Southwestern China [15]. The main vector species in Northern China is *Ixodes persulcatus* and in Southern China is *Ixodes ovatus*[15,31]; in rare cases, *Dermacentor silvarumhas* has also been identified as a carrier of TBEV [15].

In China, TBE mostly occurs sporadically from May to August, and reaches a peak during late May and early June [31]. The distribution of TBE is closely related to the distribution of the tick vectors [16]. Two natural foci for TBE exist in mainland China, the Northeast focus (Inner Mongolia, Heilongjiang, Jilin) and the Xinjiang focus [16]. Serological evidence of TBEV in 9 provinces /municipalities of Western and Southwestern China also exists (Table 1). From 1980 to 1998, 2202 cases of TBE were recorded, whereas from 1995 to 1998, only 420 infections were diagnosed [15]. Based on the statistical analysis of TBE incidence from 1952 to 1998, it appears that a peak has occurred every 5 to 7 years [31]. The TBE incidence has obvious occupational characteristics and the occupational distribution has changed significantly in recent decades. For example, the proportion of forestry workers has declined, while the proportions of farmers, students, and domestic workers have increased [32]. With the development of tourism and the disruption of forest ecological environments in recent years, the prevalence of the disease is on the rise [9].

### Crimean-Congo hemorrhagic fever

Crimean-Congo hemorrhagic fever (CCHF), also known as Xinjiang hemorrhagic fever in China, is caused by infection with a tick-borne virus (*Nairovirus*) in the family *Bunyaviridae*. It is widely distributed in Asia, Africa, and Europe, with a mortality of approximately 3-30% [33]. Its peak of incidence occurs from April to May [9]. CCHF was first described in the Crimea Peninsula of the Ukraine in 1944–1945 [34]. The virus was isolated from the blood and tissues of patients by intracerebral inoculation of suckling mice in 1967, and the virus was later shown to have the same antigenicity and biological characteristics as the Congo virus, which was isolated in 1956 from a febrile patient in Belgian Congo (now the Democratic Republic of the Congo). This led to the virus being called Crimean hemorrhagic fever-Congovirus, and later Crimean-Congo hemorrhagic fever virus [34].

Crimean-Congo hemorrhagic fever first occurred in 1965 in Bachu, Xinjiang, with 10 deaths in 11 infected patients; from 1965 to 2002, 230 cases were reported from Bachu County, with an average annual incidence of 6 [16,35]. Since 2003, no cases have been reported from Xinjiang. Another record CCHF outbreak occurred in the Junggar Basin in 1997, with 26 cases occurring within 45 days, including four deaths [35].

To date in China, CCHF cases have only been reported in Xinjiang and Jungar, while antibody-positive cases have been reported in 7 provinces /municipalities (Qinghai, Yunnan, Sichuan, Inner Mongolia, Anhui, Hainan, and Northeast Yili) (Table 1) [16]. The natural foci of CCHF are confirmed to be present in Tarim Basin, Junggar Basin, Tarim River, and the Yili River Valley border in Xinjiang province, Tengchong, Xundian, Xishuangbanna, and Menglian in Yunnan province, the Inner Mongolia Autonomous Region, as well as Sichuan, Hainan, Anhui, and Qinghai provinces [16,35].

As a disease with natural foci in deserts and pastures, CCHF is transmitted mainly by *Hyalomma asiaticum* in China, though *Ixodes spp.* may act as a carrier in some cases [35]. Sheep and hares (*Lepus yarkandensis)* in pastures in the epidemic area are its main source of infection, but patients with acute infection can also be a source; pathogens can be persist in ticks for several months and can be transmitted transovarially [9].

### Q-fever

Q-fever is an acute natural focal disease caused by the Gram-negative bacterium *Coxiella burnetii*. It was first observed in 1935 in Australia and described in 1937 [36]. Initially reported in China in 1950, Q-fever naturally spreads among wild animals (rodents) and livestock [9]. Its pathogens can persist in ticks for a long period of time and can be spread through eggs. Natural infections of *Ixodes persulcatus, Ornithodoros papillipes, Haemaphysalis campanulata, Haemaphysalis asiaticum*, *Hyalomma asiaticum kozlovi*, and *Rhipicephalus microplus* have been observed in endemic areas [9]. In recent years, new reports revealed that Q-fever may be caused by the transmission of *Coxiella burnetii* through other methods aside from vector ticks [36-39]. Unengorged ticks of the genus *Dermacentor* collected from endemic areas in Southern Germany were detected to be negative for *C. burne*tii using a specific nested PCR [37]. The same result was also reported in 887 adult *Ixode*s *ricinus* collected from 29 different localities in Southern and Central Sweden [38]. *C. burnetii* has been reported in less than 2% of *I. ricinus* in Europe [39]. Though *C. burnetii* bacteria were detected in more than 40 tick species (mainly of the genera *Ixodes*, *Rhipicephalus*, *Amblyomma*, and *Dermacentor*), *C. burnetii* is easily transmitted to healthy individuals via dust or aerosols. Thus ticks may not be a necessary vector for *C. burnetii* transmission [36]. After all, there is no good evidence that Q-fever is regularly transmitted to humans by tick-bite.

As confirmed by seroepidemiological surveys and cases, Q-fever is currently distributed in 24 provinces/municipalities in China, and outbreaks have been documented in Inner Mongonlia, Sichuan, Xinjiang, Yunnan, and Tibet (Table 1).

### Tularemia

Tularemia, caused by *Francisella tularensis*, is widely distributed, with epidemics in Europe, Asia, and North America. The first case of tularemia was observed in Tulare county, California, US in 1912 [40]. Its natural foci are limited to the Northern Hemisphere [40] In China, the causative agent was first isolated from ground squirrels in 1957, and the first case of human infection was reported in Heilongjian in 1959 [12]. Later, natural foci were reported to exist in Tibet, Xinjiang, and Gansu [12]. Tularemia cases have mainly been reported in 7 provinces/municipalities of Beijing, Heilongjiang, Inner Mongolia, Qinghai, Shandong, Tibet, and Xinjiang [10,12,13] (Table 1). In 1986, 31 cases of human infection were reported in a meat processing plant in Shandong province; since then, no further cases have been reported in China [12].

Two tick species, *Dermacentor silvarum* and *Ixodes persulatus* were reported to harbor the pathogen of tularemia (*F. tularensis subsp. Holarctica*) in the natural environment, indicating these two tick species might have a role in tularemia existence in China [10]. Rodents and wild animals are its main source of infection, and infection generally occurs in spring and early summer. Pathogens can survive in ticks for 200–700 days [12].

### North-Asia tick-borne spotted fever

North-Asia tick-borne spotted fever (NATBSF) is also known as Siberian tick-borne typhus, North Asian tick-borne rickettsiosis, North Asian tick typhus, and North Asia Fever. Its causative agent is *Rickettsia sibirica*, and small rodents are its main source of infection. This tick-borne disease was first described in 1936 in Russia [41]. Since this type of infection is common in many republics of North Asia, much effort has been invested into the studies of NATBSF [41]. An anti-*Rickettsia sibirica* antibody was first detected in the serum of humans and animals in Inner Mongolia in 1958 [42]. The first case was observed in Hulin city, Heilongjiang province in 1962, where the HL-84 strain of *Rickettsia sibirica* was first isolated from a wild rodent, *Microtus fortis*. Later, the *Rickettsia sibirica* JH-74 strain was isolated from *Dermacentor nuttalli* in 1974 in Jinghe county, Xinjiang autonomous region, and the An-84 strain was isolated from a patient in 1984 [42]. *Dermacentor nuttalli* is the main vector for North-Asia TBSF; however, *Dermacentor marginatus*, *Dermacentor sinicus*, *Derraacentor silvarum* and *Haemaphysalis yeli* can also be vectors for the disease [42]. Its pathogens can be passed through eggs and can survive for two years in ticks. Because methods for diagnosing this disease are not yet standardized, its prevalence in China has yet to be determined; however, cases have been reported in 7 provinces/municipalities of Northern Heilongjiang, Inner Mongolia, Xinjiang, Beijing, Guangdong, Jilin, and Liaoning (Table 1). The natural foci have been reported to exist in most of Northern China (at longitude 90° ~ 135° East and latitude 40° ~ 50° North); serologic clues have also been found in the serum of humans and rodents in parts of Southern China [42].

### Emerging tick-borne diseases in China

#### Human monocytic ehrlichiosis (HME)

Human monocytic ehrlichiosis (HME) is an emerging zoonosis, which was first described in the United States in 1987, and the first case of HME was documented in 1991 in the United States; the causative agent is *Ehrlichia chaffeensis*, which is an obligate intracellular pathogen affecting monocytes and macrophages [43]. Frequent symptoms of this disease are fever, chills, headache, myalgia, nausea, rash, leukopenia, thrombocytopenia, elevated serum aminotransferase levels, and elevated creatinine levels; the case-fatality rate is approximately 1.9% or higher [43].

Since the first case of HME was observed in 1999 in China, the epidemic situation of HME has been investigated in North and South China. The bacterium *Ehrlichia chaffeensis* has been detected with serological and PCR detection methods among people in Xinjiang, Inner Mongolia, Heilongjiang, Guangdong, Guangxi, Fujian, and Yunnan, 7 provinces /municipalities [44] (Table 1), and the vectors are reported to be *A. testudinarium*, *H. yeni*, *D. silvarum*[21].

### Human granulocytic anaplasmosis

Human granulocytic anaplasmosis (HGA) is another emerging tick-borne zoonosis, which was first reported in the United States in 1990 and in Europe in 1997 [45]. As the causative rickettsia was reclassified from the genus *Ehrlichia* to *Anaplasma phagocytophilum*, the disease name was changed from human granulocytic ehrlichiosis to HGA in 2001 [22,45]. The pathogen causes the disease by infecting human neutrophils [21,46]. The symptoms of HGA are similar to HME, and deaths from HGA are approximately 0.6% of those infected and typically involve those immunocompromised individuals, usually 10 or more days after disease onset [43,46].

The first case of human HGA caused by *Anaplasma phagocytophilum* was identified in Anhui in 2006, which eventually resulted in an outbreak and included 1 index case and 9 secondary infection cases probably nosocomially acquired through cutaneous or mucous membrane contact with blood or bloody respiratory secretions of the index case [45]. Such cases, including deaths, were reported in 7 P/A of Anhui, Tianjin, Shandong, Heilongjiang, Inner Mongolia, Xinjiang, and Hainan; *Ixodes persulcatus*, *Haemaphysalis longicornis*, and *Haemaphysalis concinna* are suspected as the main vectors of the disease in China, but detailed transmission evidence is still unavailable [21,23,24] (Table 1).

### Novel bunyavirus infection

A hemorrhagic fever–like illness caused by a novel bunyavirus was reported in China recently. This illness was given the name of Fever, Thrombocytopenia and Leukopenia Syndrome (FTLS) or Severe Fever with Thrombocytopenia (SFTS); the causative virus was determined to be the Huaiyangshan virus (HYSV), Henan Fever virus (HNFV), FTLS virus (FTLSV), or SFTS virus (SFTSV) [19,20,47,48].

First emerging in Henan province in 2007, and again between 2008 and 2010, cases of a life-threatening disease with sudden fever, thrombocytopenia, and leukopenia (defined as FTLS) were reported. Patients reported a history of tick bites, suggesting this disease could be infectious or tick-transmitted [47]. Many patients were previously diagnosed with human granulocytic anaplasmosis (HGA); however, only 24 of 285 (8%) had a confirmed HGA infection [47]. In this case, other pathogens aside from *Anaplasma phagocytophilum* may contribute to FTLS. A novel bunyavirus was observed in some cases, but only in clinical samples assessed with illumina sequencing. Further isolation of the virus and epidemiologic investigation confirmed that the novel bunyavirus was associated with FTLS, and was almost sequence identical (99% identity) to SFLV [19,47].

Between late March and mid-July 2009, an infectious disease emerged in rural areas of Hubei and Henan provinces; patients presented with symptoms of fever, thrombocytopenia, gastrointestinal symptoms, leukocytopenia, and the illness had an unusually high initial case fatality rate of 30% (the mortality of all infections is between 8% and 16%) [19,20]. As the disease was characterized by acute fever and thrombocytopenia, it was defined as SFTS [19]. A few months later, a novel bunyavirus was isolated from a patient’s blood during the outbreak of SFTS in Xinyang City in Henan province in 2009 [19,20].

By the end of 2011, SFTS had been reported in 11 provinces, including Henan, Hubei, Anhui, Shandong, Jiangsu, Zhejiang, Liaoning, Yunnan, Guangxi, Jiangxi, and Shaanxi (Table 1) [19,20]. As of August 2011, a total of 622 SFTS cases had been reported throughout China, mainly in Henan, Hubei, Shandong, Anhui, Liaoning, Jiangsu, and Zhejiang [1]. A number of human infection clusters have also been identified, suggesting the possibility of human-to-human transmission [47,49].

Most patients affected with SFTS lived in hilly areas or dense jungle areas and had a history of outdoor work; a small number of the patients had a history of tick bites [1,31]. Previous studies have detected SFTSV in *Haemaphysalis longicornis* and *Rhipicephalus microplus* ticks collected from a number of domestic animals, including cattle, buffalo, goats, cats and dogs [48]. Natural hosts remain to be further determined, but serological positivity has been observed in dogs, cattle, sheep, and other livestock in villages where the patients lived [1].

Many questions related to SFTS caused by the new bunyavirus remain unanswered, such as host animal, route of transmission, clinical classification, pathophysiological features, pathogenesis, and clinical treatment [1].

## Conclusions

**Figure 1:**
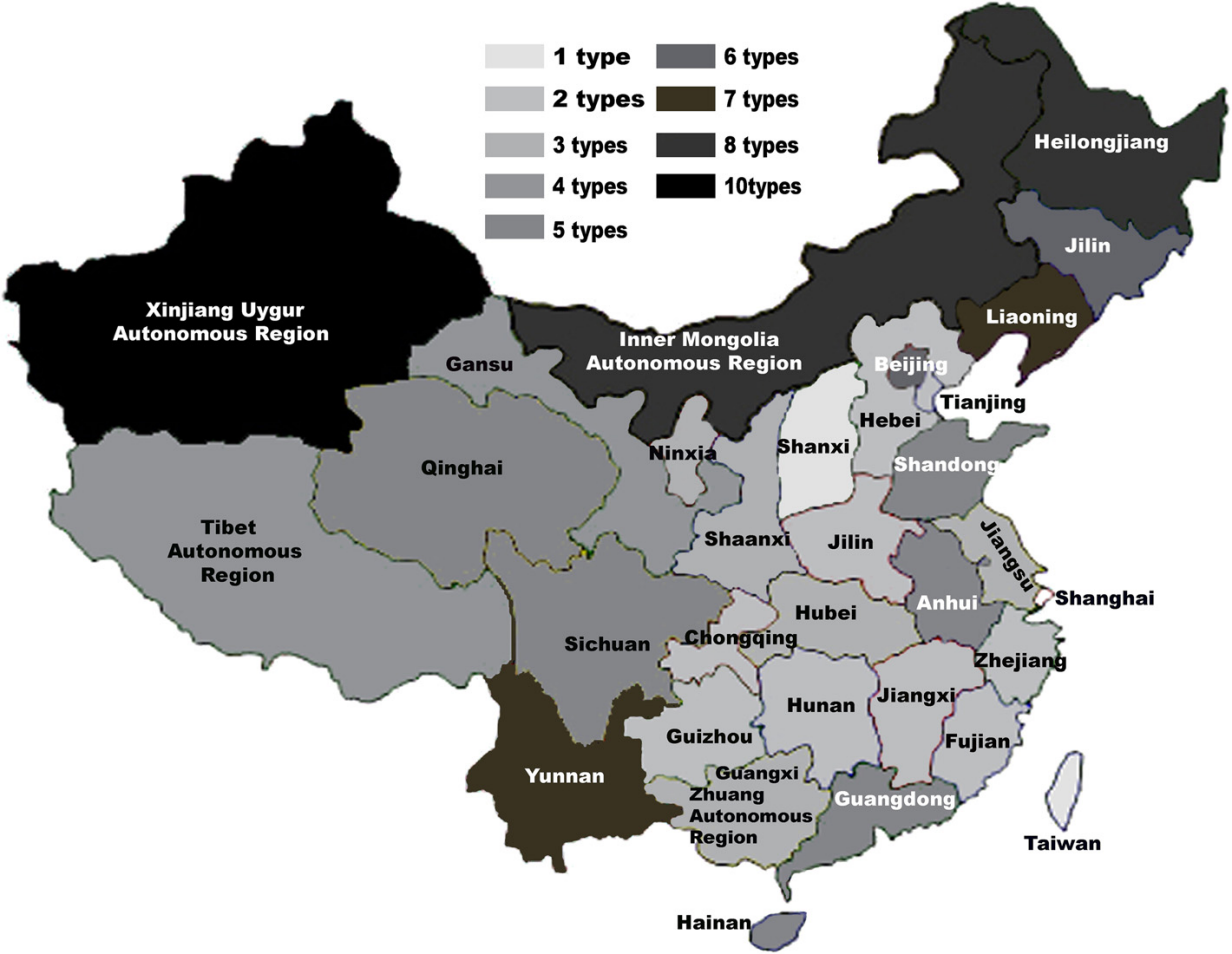
**Distribution of tick-borne diseases across China.** The tick-borne diseases are highly prevalent in North of China, including Xinjiang Uygur, Inner Mongolia, Heilongjian, Liaoning, Jilin, Beijing provinces/ autonomous regions/ municipalities (P/A/M), as well as Northeastern China including Yunnan province. The corresponding vector borne diseases reported in these P/A/M are indicated in Figure 2.

**Figure 2:**
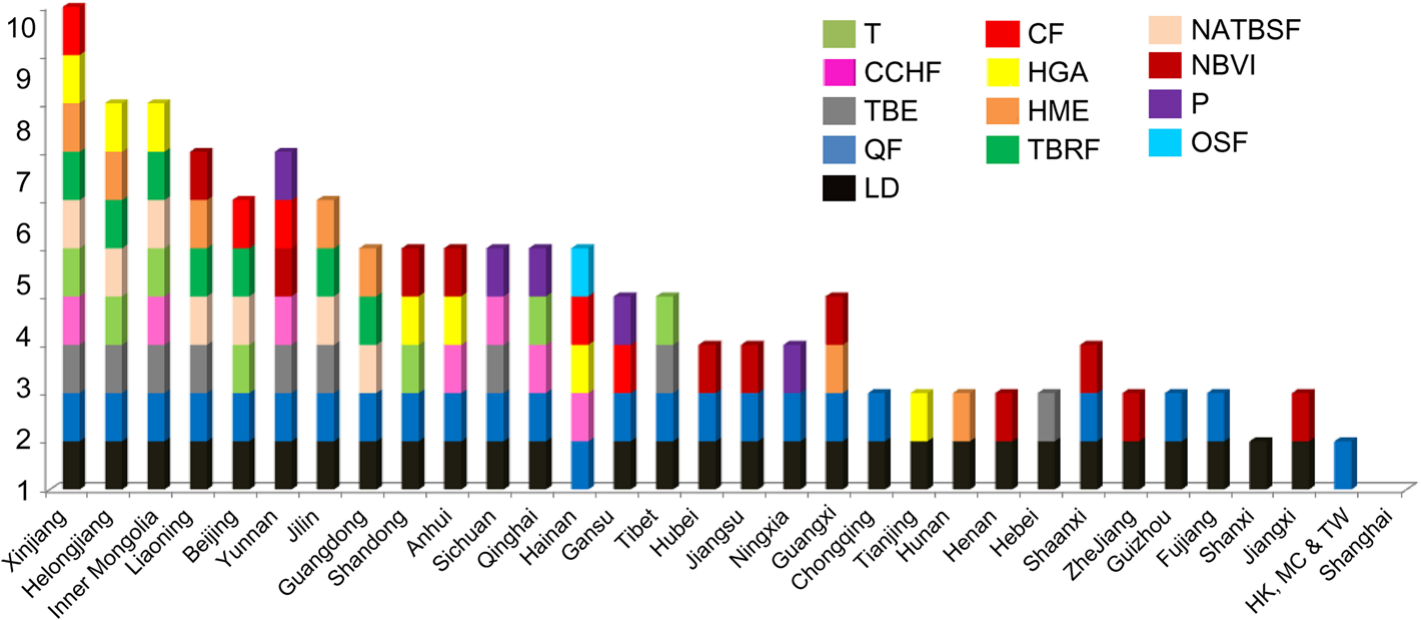
**Types of tick-borne diseases reported in China’s provinces/ autonomous regions/municipalities (P/A/M).** There are more than 13 types of tick-borne diseases are reported in different provinces/autonomous regions/municipalities in China, including TBE (tick-borne encephalitis/Forest Encephalitis in China), CCHF (Crimean-Congo hemorrhagic fever/Xinjiang hemorrhagic fever in China), CF (Colorado fever), LB (Lyme borreliosis), QF (Q-fever), T (tularemia), NATBSF (North-Asia tick-borne spotted fever), TBRF (Tick-borne relapsing fever), HGA (human granulocytic anaplasmosis), HME( human monocytic ehrlichiosis), NBVI ( Novel Bunya virus infection), P (piroplasmosis), and OSF (oriental spotted fever).

The incidence and prevalence of vector-borne diseases have regional features in spatial distribution, and the relative distribution of tick-borne diseases in China is shown in Figure 1. A high prevalence of tick-borne diseases exists in Northern China in areas such as Xinjiang, Inner Mongolia, Heilongjiang, Liaoning, Jilin, and in Northeastern China in places like Yunnan. The major tick-borne diseases in high-incidence areas include Lyme borreliosis, Q-fever, and TBE (Figure 2). Other tick borne diseases that are not frequently reported in China include Colorado fever, piroplasmosis, and oriental spotted fever (Table 1).

Several reasons for the wide dissemination of tick-borne diseases throughout China exist. First, ticks of numerous species are widely distributed, with diverse living habits and numerous hosts, including birds, reptiles, and mammals. Secondly, the vast territory, complex geography, climate variability, and diverse ecological environments in China provide various habitats for ticks. Thirdly, the rapid development of international and inter- regional exchange has created favorable conditions for the spread of tick-borne diseases. Finally, the epidemic area of tick-borne disease is gradually expanding along with changes and ecological damage of the forest environment.

Continuous efforts are still required for the prevention and treatment of tick-borne diseases in China. Distribution of all species of ticks across China should be investigated as thoroughly as possible, so that targeted destruction of ticks can be carried out during their active seasons. Research on the rapid diagnosis of tick-borne diseases and mechanisms of pathogen-transmission needs to be enhanced. More effective drugs and vaccines against tick-borne diseases are needed and new tick prevention methods must be developed. Finally, integrated biological control of ticks will bring efficient, high-speed, long-term, and pollution-free effects in China.

## Competing interests

None declared.

## Authors’ contributions

X-BW: Manuscript drafting. R-HN: Data collection and manuscript drafting. S-SW: Data collection and figure creation. J-SZ: Manuscript proofreading. H-JP: Manuscript drafting, data proofing and manuscript submission. All authors read and approved the final version of the manuscript.
